# Hypofractionated Radiotherapy Dose Scheme and Application of New Techniques Are Associated to a Lower Incidence of Radiation Pneumonitis in Breast Cancer Patients

**DOI:** 10.3389/fonc.2020.00124

**Published:** 2020-02-11

**Authors:** Byung Min Lee, Jee Suk Chang, Se Young Kim, Ki Chang Keum, Chang-Ok Suh, Yong Bae Kim

**Affiliations:** ^1^Department of Radiation Oncology, Yonsei University College of Medicine, Seoul, South Korea; ^2^Department of Radiation Oncology, CHA Bundang Medical Center, CHA University, Bundang-gu, South Korea

**Keywords:** radiation pneumonitis, radiotherapy, breast cancer, hypofractionation, lung dosimetry

## Abstract

**Purpose:** Radiation pneumonitis (RP) is one of the most severe toxicities experienced by patients with breast cancer after radiotherapy (RT). RT fractionation schemes and techniques for breast cancer have undergone numerous changes over the past decades. This study aimed to investigate the incidence of RP as a function of such changes in patients with breast cancer undergoing RT and to identify dosimetric markers that predict the risk of this adverse event.

**Methods and Materials:** We identified 1,847 women with breast cancer who received adjuvant RT at our institution between 2015 and 2017. The RT technique was individually tailored based on each patient's clinicopathological features. Deep inspiration breath hold technique or prone positioning were used for patients who underwent left whole-breast irradiation for cardiac sparing, while those requiring regional lymph node irradiation underwent volumetric-modulated arc therapy (VMAT).

**Results:** Of 1,847 patients who received RT, 21.2% received the conventional dose scheme, while 78.8% received the hypofractionated dose scheme (mostly 40 Gy in 15 fractions). The median follow-up period was 14.5 months, and the overall RP rate was 2.1%. The irradiated organ at risk was corrected concerning biologically equivalent dose. The ipsilateral lung V_30_ in equivalent dose in 2 Gy (EQD2) was the most significant dosimetric factor associated with RP development. Administering RT using VMAT, and hypofractionated dose scheme significantly reduced ipsilateral lung V_30_.

**Conclusions:** Application of new RT techniques and hypofractionated scheme significantly reduce the ipsilateral lung dose. Our data demonstrated that ipsilateral lung V_30_ in EQD2 is the most relevant dosimetric predictor of RP in patients with breast cancer.

## Introduction

Radiation pneumonitis (RP) is one of the most severe toxicities caused by radiotherapy (RT) in patients with breast cancer. Although sometimes fatal, particularly in elderly patients or those with medical comorbidities, most symptoms of RP can be resolved with steroid-based medications. In the era of two-dimensional conventional RT, the central lung distance, defined as the distance between the midpoint of the posterior field and the chest wall, was used as an indicator of RP (1, 2). After the introduction of treatment planning based on three-dimensional conformal RT (3D CRT), studies have aimed to identify the dosimetric parameters of the lung that predict RP following RT for breast cancer. However, such studies remain inadequate for clinical utility (3, 4).

There has been an important paradigm shift in RT for breast cancer over the past 20 years. First, hypofractionated dose scheme emerged as a new standard treatment for this disease. Several prospective randomized trials demonstrated that the hypofractionation RT is non-inferior to conventional fractionated RT with respect to treatment outcomes and toxicities after breast conserving surgery. Although hypofractionation RT after mastectomy is not standard of care yet, recent prospective trial has shown non-inferior results compared to conventional RT (5). More radiation oncologists have adopted such abbreviated RT methods because of their convenience and cost-effectiveness (6–9). Along with hypofractionated RT, some other techniques that have become available include volumetric-modulated arc therapy (VMAT), deep inspiration breath-hold (DIBH), and prone positioning.

In this study, we aimed to investigate the incidence of RP and identify the dosimetric markers that predict the risk of this adverse effect as a function of changes in hypofractionated dose schemes and application of new RT techniques used to treat breast cancer.

## Materials and Methods

### Patients

We identified patients who underwent adjuvant RT following surgery for breast cancer at our institution between January 2015 and December 2017 using a prospectively collected registry (*n* = 2,130). We excluded patients who had distant metastases at the time of their diagnosis (*n* = 42), those who did not visit at regular follow-up (*n* = 114), and those who were followed at other hospitals (*n* = 127). Finally, 1,847 patients who met the eligibility criteria were included in our study cohort. We reviewed the medical charts of all patients to determine the incidence of RP. This study was approved by the institutional review board of Severance hospital (4-2018-0663).

### Treatment

We performed computed tomography (CT) simulation (SOMATOM sensation; Siemens, Erlangen, Germany) with 3 mm-thick slices for all patients. For immobilization, patients positioned their ipsilateral arms in abduction and used a thermoplastic immobilization system (Type-S; Medtec, Alton, IA, USA). Per our institutional protocol, the irradiation technique was optimized for each individual to minimize the dose to the heart while maximizing target dose homogeneity. Patients with large, pendulous, or ptotic breasts were placed in the prone position to avoid skin reactions at the inframammary fold. For cancer of the left breast, the DIBH technique was applied to displace the heart from the chest wall, as described previously (10); patients were instructed to apply the Abches breathing monitoring device (APEX Medical, Tokyo, Japan) during DIBH. If the distance between the heart and chest wall was sufficient to lower the heart dose using DIBH by inflating the lung volume, we performed RT using the DIBH technique. Due to the setup uncertainties in prone positioning, we underwent daily cone beam CT during RT. However, if the heart was not sufficiently spared by DIBH or if internal mammary node (IMN) irradiation (IMNI) was required, we performed RT with VMAT for cardiac sparing.

For 3D CRT, target volumes were delineated based on palpating breast tissue and adding a margin; the border of the intact breast and treatment planning for 3D CRT was specified as described elsewhere (11). Regional lymph node irradiation (RNI), including that of the internal mammary, axillary, and supraclavicular lymph nodes, was recommended to patients with metastatic nodes or those with high-risk N0 breast cancer (i.e., tumor sizes larger than 2 cm, high-grade tumors, and estrogen receptor-negative tumors) based on two large scale randomized trials (12, 13). The partial wide tangent field technique was used to cover the entire breast as well as the IMNs. The supraclavicular and axillary lymph nodes were irradiated using a separate beam that did not overlap with that of the breast field. In patients who had undergone mastectomy, the chest wall and regional nodes were irradiated using the reverse hockey stick technique as described elsewhere (14). Since June 2015, we performed hypofractionation in patients who received mastectomy.

For VMAT, target volumes and organs at risk were contoured based on European Society for Radiotherapy and Oncology guidelines, which was validated using both single-center and multi-center datasets in Korea (15). For patients with T4 stage or N2-N3 stage, we followed the Radiation Therapy Oncology Group breast cancer target guidelines. For VMAT planning, two partial arcs were used limiting the unnecessary arc segments without compromising dose quality. Plan generation and dose calculation were performed using the RayStation treatment planning system (version 5.0, RaySearch, Stockholm, Sweden). For treatment, 6 MV photon beams emitted from a linear accelerator (Versa HD, Elekta, Stockholm, Sweden) were used. The 95% isodose encompassed the entire planning target volume, and volumes in target areas receiving over 107% of the prescribed dose were minimized. The planning requirements for organ at risk were as follows: ipsilateral lung V_5_ <50%, V_10_ <35%, V_20_ <20% (V_χ_ defined as the percentage of the total volume exceeding χ Gy), mean heart dose <3 Gy, mean left coronary artery dose <6 Gy (maximum point dose [Dmax] <10 Gy), mean contralateral breast dose <2 Gy, esophagus Dmax <12 Gy, and mean thyroid dose <3 Gy. We concerned the esophagus and thyroid to reduce radiation induced esophagitis and hypothyroidism. Cone-beam CT images were obtained daily to verify appropriate patient set-up and minimize positioning errors. During the study period, three different fractionation schedules were used: either 40.05 Gy in 15 fractions (*n* = 1,055, 57.1%), or 42.56 Gy in 16 fractions (*n* = 400, 21.7%) for hypofractionation and 50.4 Gy in 28 fractions (*n* = 392, 21.2%) schedule for conventional fractionation.

For tumor bed boost, 9 Gy in 5 fractions was applied in conventional fractionation (*n* = 297, 20.8%). The tumor bed boost in hypofractionated RT differed depending on RT modalities. In case that patients received RT using 3D CRT in hypofractionation, 10 Gy in 5 fractions was applied (*n* = 541, 37.9%). The electron beams were used for boost with 3D CRT. For patients treated by VMAT, the tumor bed boost was performed using simultaneous integrated boost. The simultaneous integrated boost dose was determined based on RTOG 1005 protocol. Total dose of 48 Gy in 15 fractions was applied to tumor bed while total dose of 40.05 Gy in 15 fractions was given to the whole breast or whole breast plus regional LN (*n* = 373, 26.2%).

### Analysis

The primary endpoint was the occurrence of symptomatic RP, defined as respiratory symptoms (e.g., dyspnea, non-productive cough) with correlated radiologic images (e.g., chest radiography and CT). The RP was graded using common terminology criteria for adverse events version 5.0. Radiation oncologists prescribed oral prednisolone until symptoms were relieved. To evaluate the factors affecting the occurrence of RP, univariate and multivariate analyses using Cox proportional hazards models were performed. In multivariate analysis, the factors significant (*p* < 0.05) in univariate analysis were used. The factors related to RP in other studies were also included for multivariate analsysis. The receiver operating characteristic (ROC) and area under the curve analyses were used to identify the optimal cutoff values that best predict the occurrence of RP. The comparison between hypofractionation group and conventional fractionation group was performed using chi-squared test. The logistic regression analysis was used to evaluate the factors associated with the lung dose parameters.

For dosimetric analysis, the planning data of all the patients were transferred into the MIM software (version 6.7.14; Cleveland, OH, USA) for multiple-plan comparison. To analyze the ipsilateral lung dose parameter, we collected the ipsilateral mean lung dose, V_5_, V_10_, V_15_, V_20_, V_30_, and V_40_. The ipsilateral lung dose parameters were converted into equivalent dose in 2 Gy (EQD2) with α/β ratio of 3 Gy to correct for hypofractionation. All tests were conducted by using either the SPSS software version 20.0 (IBM Corp., Armonk, New York, USA) or R version 3.3.2 (R Foundation for Statistical Computing, Vienna, Austria).

## Results

### Patient Characteristics

The patient and tumor characteristics are summarized in Table 1. Approximately 85% of the patients had early breast cancer (stages 0–2). In total, 38.5% received RNI and 51.9% received either neoadjuvant or adjuvant chemotherapy. Adjuvant RT was performed either via the conventional dose scheme (21.2%) or the hypofractionated dose scheme (78.8%); 44.7% of patients underwent adjuvant RT using VMAT.

**Table 1:** Patient characteristics and treatment characteristics.

			**CF group**		**HF group**		
	**No. of patients**	**%**	**No. of patients**	**%**	**No. of patients**	**%**	***p***
Age (Year)							0.41
<51	908	49.2%	200	51.0%	708	48.7%	
≥51	939	50.8%	192	49.0%	747	51.3%	
Pathology							
Ductal carcinoma *in situ*	254	14.1%	59	15.1%	195	13.4%	0.56
Invasive ductal carcinoma	1,327	71.7%	283	72.2%	1,044	71.8%	
Invasive lobular carcinoma	85	4.6%	12	3.1%	71	4.9%	
Mucinous carcinoma	36	1.9%	15	3.8%	21	1.4%	
Tubular carcinoma	43	2.3%	10	2.6%	33	2.3%	
Stage							0.08
0	261	14.5%	55	14.0%	206	14.2%	
I	740	40.1%	171	43.6%	569	39.1%	
II	592	31.8%	110	28.1%	482	33.1%	
III	253	13.6%	56	14.3%	197	13.5%	
Surgery							0.01
Breast conserving mastectomy	1,485	80.4%	297	75.8%	1,188	81.6%	
Mastectomy	362	19.6%	95	24.2%	267	18.4%	
Lung disease^*^							0.94
Yes	9	0.5%	2	0.5%	7	0.5%	
No	1,838	99.5%	390	99.5%	1,488	99.5%	
Smoking history							0.45
Yes	73	4.2%	13	3.5%	60	4.4%	
No	1,728	95.8%	358	96.5%	1,370	95.6%	
Regional LN irradiation							0.08
Yes (SCL+IMN+AXL)	712	38.5%	166	42.3%	546	37.5%	
No	1135	61.5%	226	57.7%	909	62.5%	
Chemotherapy							0.52
Yes							
Neoadjuvant CTx	402	21.8%	89	22.7%	313	21.5%	
Adjuvant CTx	557	30.2%	109	27.8%	448	30.8%	
No	888	48.1%	194	49.5%	694	47.7%	
Hormone therapy							0.73
Yes	1,297	70.2%	278	70.9%	1,019	70.0%	
No	550	29.8%	114	29.1%	436	30.0%	
RT technique							<0.001
Free-breathing	1,258	68.1%					
FIF	226	12.2%	11	2.8%	215	14.8%	
Wedge	194	10.5%	178	45.4%	16	1.1%	
RHT	13	0.7%	13	3.3%	0	0.0%	
VMAT	825	44.7%	8	2.0%	817	56.2%	
DIBH	488	26.4%					
FIF	322	17.4%	22	5.6%	300	20.6%	
Wedge	164	8.9%	153	39.0%	11	0.8%	
RHT	2	0.1%	2	0.5%	0	0.0%	
Prone	101	5.5%					
FIF	97	5.3%	4	1.0%	93	6.4%	
Wedge	4	0.2%	1	0.3%	3	0.2%	

* *COPD, ILD were included*.

*CF, Conventional fractionation; HF, Hypofractionation; SCL, Supraclavicular lymph node; IMN, Internal mammary lymph node; AXL, Axillary lymph node; CTx, Chemotherapy; FIF, Field in field; RHT, Reverse hockey stick; VMAT, Volumetric-modulated arc therapy; BCS, Breast-conserving surgery*.

We also compared the patient and tumor characteristics between the conventional fractionation group and hypofractionation group (Table 1). Most of the variables were well-balanced between two groups except for the method of surgery and the techniques used for RT. More patients in the hypofractionated group received breast conserving surgery. Also, most of the patients treated with VMAT underwent hypofractionated RT.

### RP Incidence

RP occurred in 40 patients (2.1%) within a median follow-up period of 14.5 months. The commonest symptom was a mild dry cough; few patients also experienced other symptoms such as shortness of breath. Patients experiencing RP symptoms were prescribed steroids, following which these symptoms resolved. None of the patients developed RP grade ≥3. Symptomatic RP occurred no sooner than 3 months and no later than 12 months after commencing RT.

### Comparison of Lung Dosimetry

**Figure 1:**
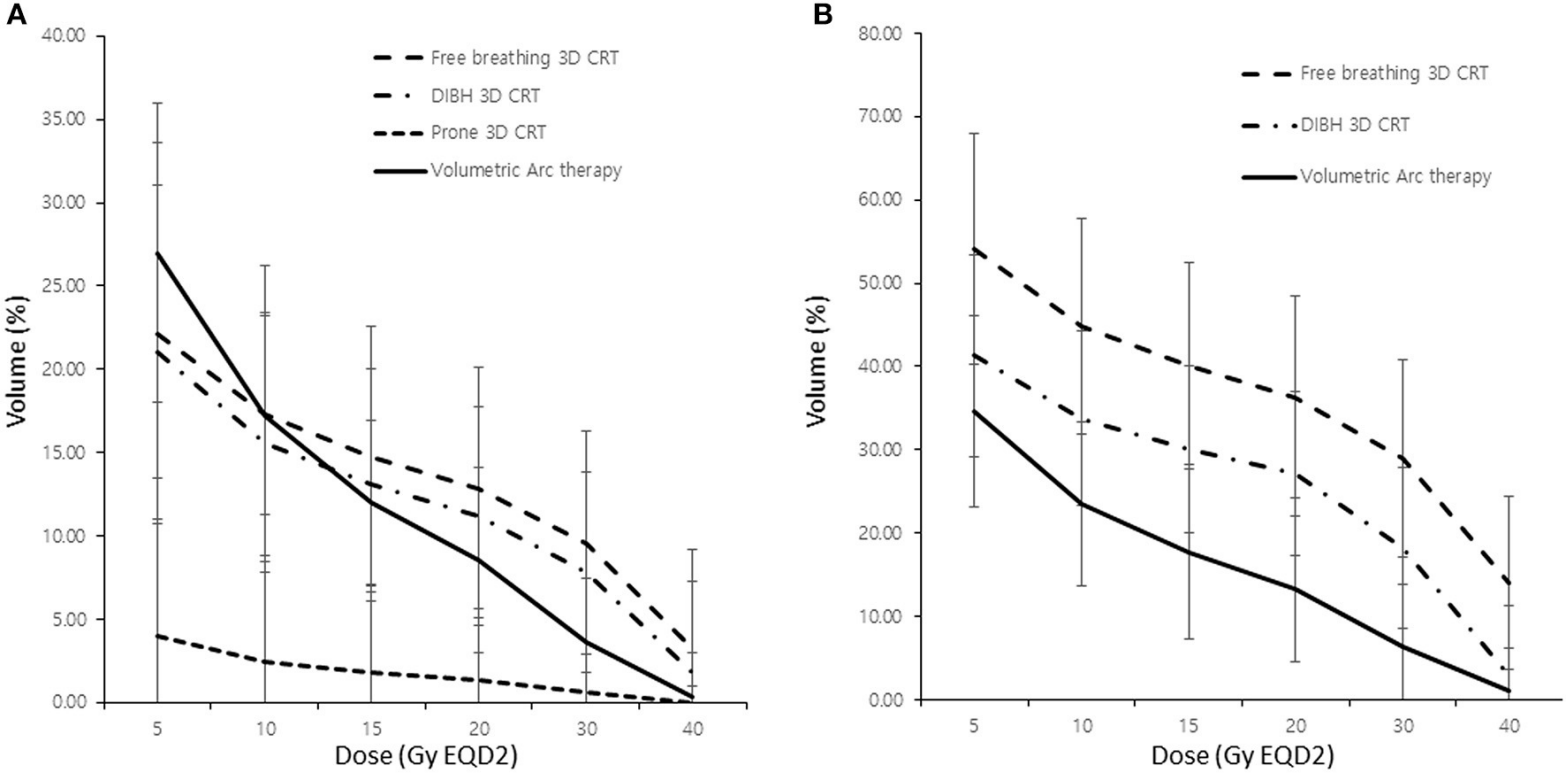
Comparison of lung dose according to the radiotherapy modality without **(A)** and with **(B)** internal mammary lymph node irradiation. 3D CRT, three-dimensional conformal radiotherapy; DIBH, deep inspiration breath hold; V_χ_, percentage of the total volume exceeding χ Gy.

The lung dosing parameters when using different RT techniques (i.e., free-breathing 3D-CRT, DIBH 3D-CRT, prone positioning RT, and VMAT) were compared (Table 2). Patients who underwent IMNI had higher doses to the lung than those who did not. The DIBH technique produced lower lung doses than the free-breathing technique especially when IMNI was performed. Among the various techniques, the lung doses in patients who used prone positioning techniques were significantly lower than that in patients using other techniques. In patients who did not undergo IMNI, the lung V_30_ and V_40_ were significantly lower in patients undergoing VMAT than in those in patients undergoing other techniques, while the lung V_5_ was higher in patients undergoing VMAT (Figure 1A). For patients who did undergo IMNI, the VMAT group showed the lowest mean lung dose (Figure 1B).

**Table 2:** Comparison of lung dose parameter depending on internal mammary node irradiation and radiotherapy technique.

	**IMN (–)**				**IMN (+)**		
	**Free-breathing 3D CRT**	**DIBH 3D CRT**	**Prone 3D CRT**	**Volumetric arc therapy**	**Free-breathing 3D CRT**	**DIBH 3D CRT**	**Volumetric arc therapy**
No. of patients	307	304	101	423	130	184	398
Mean lung dose							
Median	6.50	5.71	1.16	5.70	16.24	11.20	7.56
IQR	4.64–8.32	4.30–8.22	0.52–2.51	4.92–6.76	11.24–19.82	8.9–15.24	6.73–8.33
V_5_							
Median	22.17	21.01	4.03	26.99	54.11	41.30	34.62
IQR	16.54–30.7	15.9–30.00	0.88–8.05	22.92–31.14	43.32–62.21	35.67–51.58	30.91–38.28
V_10_							
Median	17.34	15.60	2.47	17.24	44.83	33.75	23.49
IQR	12.81–21.54	12.38–21.2	0.33–6.00	14.50–20.40	32.74–51.04	27.95–41.18	20.75–26.55
V_15_							
Median	14.79	13.07	1.77	12.00	40.09	30.10	17.76
IQR	10.94–18.42	10.15–17.88	0.17–4.54	9.95–15.08	27.91–45.49	23.19–37.02	15.40–20.08
V_20_							
Median	12.86	11.20	1.35	8.54	36.35	27.13	13.33
IQR	9.38–16.42	8.38–15.78	0.07–3.74	6.69–11.25	24.80–42.00	19.70–33.69	11.19–15.30
V_30_							
Median	9.59	7.85	0.66	3.60	29.02	18.26	6.30
IQR	6.33–13.09	5.19–12.08	0.00–2.00	2.10–5.62	15.00–35.34	11.25–24.62	4.37–8.45
V_40_							
Median	3.26	1.85	0.03	0.36	14.00	2.95	1.13
IQR	0.83–8.56	0.18–7.23	0.00–0.37	0.05–1.16	2.10–22.43	0.87–11.70	0.60–2.14

*IMN, Internal mammary lymph node; 3D CRT, 3-Dimensional conformal radiation therapy; DIBH, Deep inspiration breath-hold; VMAT, Volumetric-modulated arc therapy; IQR, Interquartile range*.

**Figure 2:**
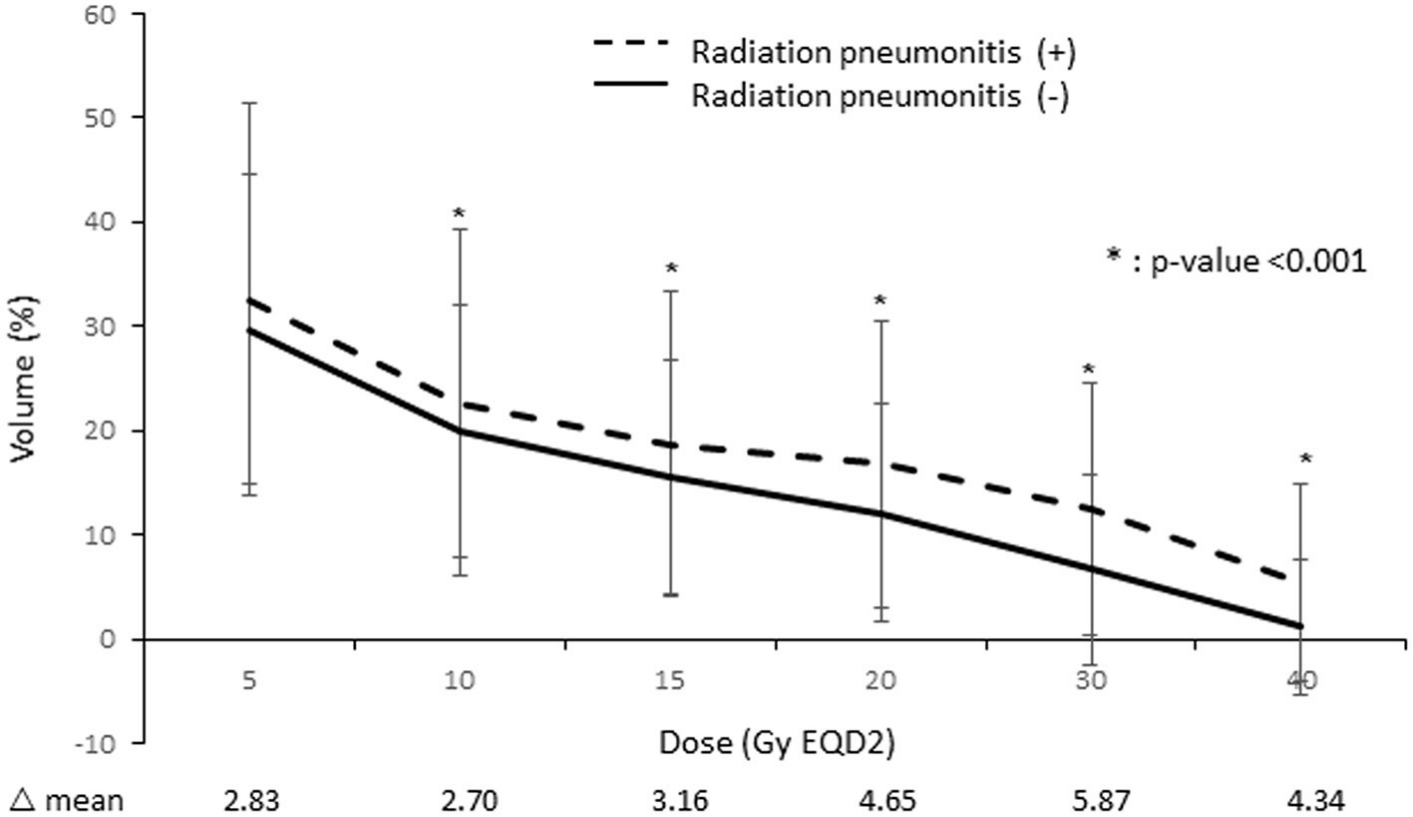
Comparison of the lung dose-volume histogram between patients who developed radiation pneumonitis and those who did not.

Moreover, we found that patients with RP showed higher dose-volume histogram parameter values in all areas than those without RP. Among these individual parameters, ipsilateral lung V_30_ showed the largest difference between these two patient groups (Figure 2).

### RPA

**Figure 3:**
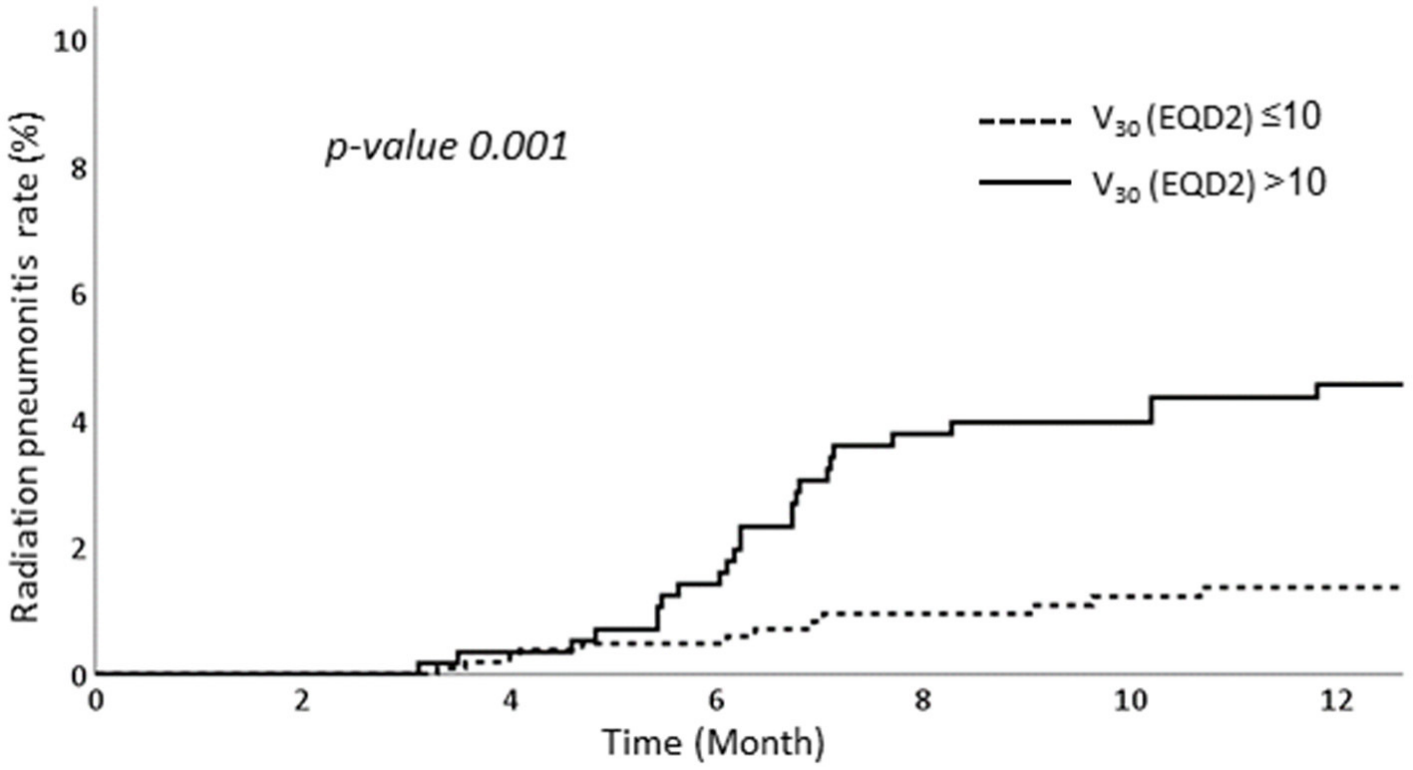
Occurrence of radiation pneumonitis according to subgroups with V_30_ >10% and V_30_≤10%.

We performed RPA to determine the factors associated with RP. Among various dosimetric parameters, the ipsilateral lung V_30_ in EQD2 >10% was associated with significantly higher RP rates than those of ipsilateral lung V_30_ in EQD2 ≤ 10%. The RP occurred in 4.6% in patients with ipsilateral lung V_30_ more than 10% while only 1.4% of patients experienced RP when ipsilateral lung V_30_ was <10% (Figure 3).

### Dosimetric Analysis

**Figure 4:**
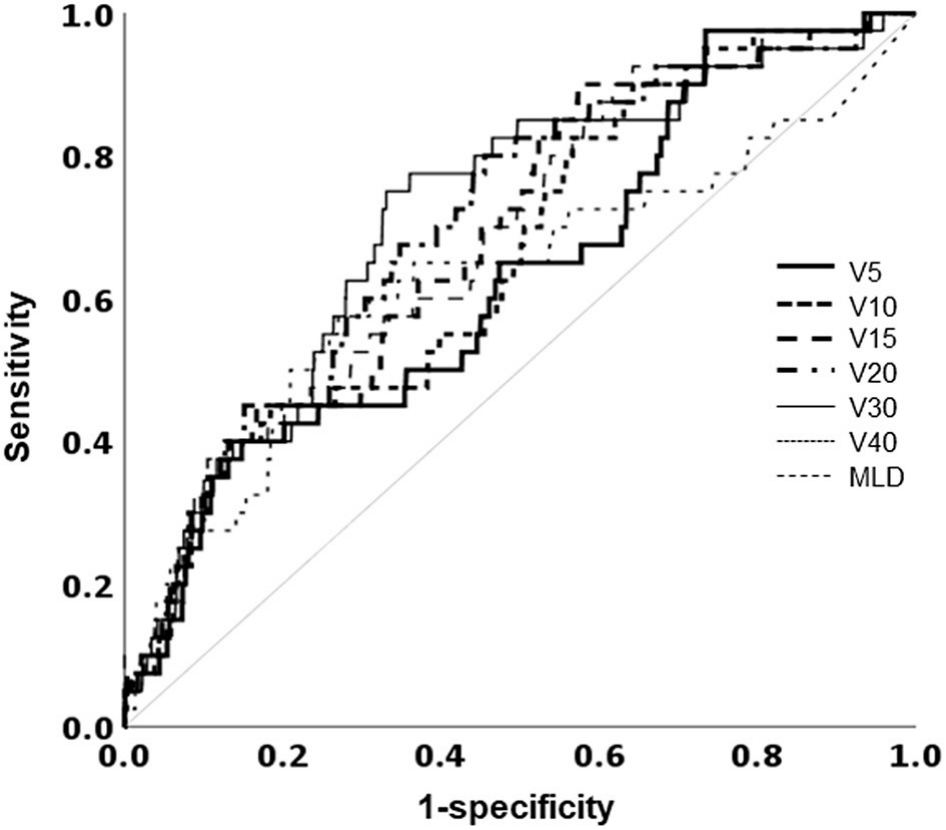
Receiver operating characteristic curve analysis for the optimal cutoff value to predict the occurrence of radiation pneumonitis. MLD, mean lung dose; V_χ_, percentage of the total volume exceeding χ Gy.

The mean lung dose, ipsilateral lung V_5_, V_10_, V_15_, V_20_, V_30_, and V_40_ were all significantly associated with RP. The largest area under the ROC curve was that of the ipsilateral lung V_30_ (Figure 4). Univariate analysis showed that hormone treatment, fractionation schedule, RT technique, and the ipsilateral lung V_30_ significantly affected RP. On multivariate analysis, patients with ipsilateral lung V_30_ larger than 10% had a significantly higher rate of RP than those with ipsilateral lung V_30_ <10% (Table 3).

**Table 3:** Univariate and multivariate analysis of factors associated with symptomatic radiation pneumonitis.

	**Univariate analysis**			**Multivariate analysis**	
	**Radiation pneumonitis rate**	**HR (95% CI)**	***p***	**HR (95% CI)**	***p***
Age (≥51 vs. <51)	1.8 vs. 2.5%	0.69 (0.37–1.29)	0.25		
Lung disease (Yes vs. No)	11.1 vs. 2.1%	5.41 (0.74–39.45)	0.09	5.90 (0.79–43.55)	0.082
Smoking history (Yes vs. No)	0 vs. 2.1%	0.05 (0.00–86.13)	0.42		
Chemotherapy (Yes vs. No)	1.9 vs. 2.5%	0.90 (0.63–1.29)	0.58		
Chemotherapy regimen			0.09		
Taxane based vs. Adriamycin based	1.1 vs. 2.4%	0.52 (0.15–1.88)	0.32		
Herceptin based vs. Adriamycin based	6 vs. 2.4%	2.06 (0.83–5.13)	0.12		
Hormone therapy (Yes vs. No)	1.7 vs. 3.3%	0.50 (0.27–0.94)	0.03	0.53 (0.28–1.01)	0.053
Regional LN irradiation (Yes vs. No)	2.4 vs. 2.0%	1.12 (0.59–2.09)	0.73		
Fraction schedule (Hypofractionation vs. Conventional fractionation)	1.5 vs. 4.6%	0.43 (0.23–0.80)	<0.01	0.63 (0.31–1.28)	0.203
Ipsilateral lung dose (V_30_ > 10% vs. V_30_ ≤ 10%)	4.6 vs. 1.4%	2.93 (1.53–5.62)	<0.01	2.89 (1.51–5.54)	0.002
RT technique			0.007		
DIBH 3D CRT vs. Free-breathing 3D CRT	2.5 vs. 5.0%	0.47 (0.23–0.95)	0.04		
Prone 3D CRT vs. Free-breathing 3D CRT	0 vs. 5.0%	NR			
VMAT vs. Free-breathing 3D CRT	0.7 vs. 5.0%	0.22 (0.09–0.55)	0.001		

*CI, Confidence interval; HR, Hazard ratio; LN, Lymph node; 3D CRT, 3-dimensional conformal radiotherapy; DIBH, Deep inspiration breath hold; VMAT, Volumetric modulated arc therapy; NR, Not reported*.

Among the RT techniques, fractionation schemes, and IMN irradiation, VMAT (odds ratio 0.12, 95% CI 0.08–0.17) was a major determinant of lowering ipsilateral lung V_30_ followed by hypofractionation (odds ratio 0.14, 95% CI 0.10–0.19). The IMN irradiation was the only factor increasing the ipsilateral lung V_30_ (odds ratio 6.59, 95% CI 4.88–8.92) (Table 4).

**Table 4:** Analysis the factor determining the lung V_30_.

	**Univariate analysis**		**Multivariate analysis**	
	**OR (95% CI)**	***p***	**OR (95% CI)**	***p***
IMN irradiation (Yes vs. No)	2.63 (2.15–3.22)	<.001	6.59 (4.88–8.92)	<.001
Hypofractionation (Yes vs. No)	0.07 (0.06–0.10)	<.001	0.14 (0.10–0.19)	<.001
VMAT (Yes vs. No)	0.12 (0.10–0.16)	<.001	0.12 (0.08–0.17)	<.001
Prone (Yes vs. No)	NR	NR		
DIBH (Yes vs. No)	0.30 (0.24–0.38)	<.001	0.98 (0.73–1.33)	0.911

*OR, Odds ratio; CI, Confidence interval; IMN, Internal mammary node; VMAT, Volumetric modulated arc therapy; DIBH, Deep inspiration breath hold; NR, Not reported*.

## Discussion

This study investigated the incidence of RP in patients with breast cancer who underwent conventional and hypofractionation RT and identified dosimetric markers that predict the risk of RP. We demonstrated that ipsilateral lung V_30_ is the dosimetric predictor of RP that is the most relevant in patients with breast cancer. The change in RT techniques using VMAT and hypofractionation dose schemes reduce the ipsilateral lung V_30_.

Several studies have shown that the occurrence of RP is affected by both patient-related and treatment-related factors. Patient-related factors that affect the incidence of RP include existing lung disease, poor pulmonary function, and smoking history (16, 17). Meanwhile, treatment-related factors known to affect RP development in patients with lung cancer include radiation dose, irradiated lung volume, schedule of fractionation, and usage of chemotherapy (18–21). However, as patients with breast cancer tend not to have underlying lung diseases or smoking histories with the same frequencies as those with lung cancer, our study revealed no association between patient-related factors and the occurrence of RP. However, treatment-related factors, particularly those related to RT, did affect RP development, as reported previously (11).

In this study, we showed that the hypofractionation dose scheme lowered the ipsilateral lung dose V_30_. It was suggested that the α/β ratio for breast tissue ranges from 3 to 4 Gy, which is similar to that of normal tissues (6), and this was later confirmed in the START A, START B, and another Canadian study (8, 22, 23). Hence, hypofractionation has become the standard treatment for breast cancer. While hypofractionation did not significantly affect RP development in our study, the V_30_ of the ipsilateral lung were found to be strong predictors of RP.

We also demonstrated that advances in RT techniques have reduced the incidence rate of RP. Improvements that were designed to reduce the dose to the heart while maintaining RT safety and efficacy include intensity-modulated RT (IMRT), DIBH, and prone positioning; these techniques also significantly decreased the dose to the lung by more than 50%. The DIBH can lower the dose to the heart, but the coverage of the ipsilateral whole breast planning target volume can be suboptimal. In case that the tumor was located at medial location of the breast, tangent fields are difficult to fully cover tumor bed and avoid the heart simultaneously. Even though the prone positioning for breast enables lowering the dose to the lung and heart, but setup uncertainties exist for prone positioning (24). By contrast, IMRT made it possible to protect the heart and ipsilateral lung without compromising target coverage and set up uncertainties.

Because landmark studies such as the MA 20 and EORTC 22922 trials demonstrated that RNI can reduce the risk of early breast cancer recurrence (12, 13), radiation oncologists increasingly consider its application but remain hesitant owing to the risk of toxicity to the heart and lung. While we perform DIBH and prone positioning for patients at our hospital who are not undergoing RNI, 3D CRT with partially wide tangent fields has been performed in patients requiring RNI, including IMNI. IMRT for breast cancer is widely used today after it became reimbursable by the national insurance program in our country in 2015. Our study showed that IMRT can sufficiently cover the whole breast and regional lymph nodes, particularly IMNs, while effectively reducing lung, and heart toxicity.

The chemotherapy regimen did not affect the incidence of RP in our study. As some chemotherapeutic agents act as sensitizers to radiation, the patients who received chemotherapy could be at higher risk to RP. The article showing that chemotherapy increased the risk of RP demonstrated that sequential chemotherapy diminished the risk of RP as compared to concurrent chemoradiotherapy (25). In our study, none of the patients underwent concurrent chemotherapy during RT. As sequential chemotherapy has minimal impact on development of RP, neither the chemotherapy regimen nor the use of chemotherapy increased the risk of RP in our study.

Sequential tumor bed boost was applied in patients treated with 3D CRT while simultaneous integrated boost was used in patients treated with VMAT. In this study, sequential tumor bed boost dose was not accounted for analysis. However, as electron beams were used for sequential tumor bed boost in case of patients treated with 3D CRT, we believed that the effect of tumor bed boost to the lung dose was negligible.

No significant parameters predicting the occurrence of RP in patients with breast cancer have been identified to date. The 3D CRT technique can reduce the areas receiving low irradiation doses (e.g., the V_5_ and V_10_) on the dose-volume histograms but not the areas receiving high doses. By contrast, the VMAT technique can reduce the areas of high RT dose while widening the areas of low irradiation (26). Previous studies in patients with breast cancer showed that V_20_ lung constraints could markedly reduce RP (27, 28). However, our results also demonstrated that V_30_ constraints were significantly associated with reduced RP rates in patients with breast cancer.

Our study was limited by its retrospective design and single-institution analysis. Some unbalance existed in patient characteristics between hypofractionated and conventional fractionated RT group, as the surgical method and RT techniques were significantly different between two groups. Also, the number of patients with RNI and without RNI differ largely. Although most of the factors were well-balanced between two groups, careful interpretation of results is needed. As such, external validation is necessary to confirm our findings.

In conclusion, our study demonstrated that the hypofractionation dose scheme and RT techniques such as VMAT can reduce the radiation dose and potentially the incidence of RP. Although external validation is still required, we clearly showed that ipsilateral lung V_30_ in EQD2 is reliable dosimetric predictors of RP in patients with breast cancer.

## Data Availability Statement

The datasets generated for this study are available on request to the corresponding author.

## Ethics Statement

The studies involving human participants were reviewed and approved by institutional review board of Severance hospital (4-2018-0663). Written informed consent for participation was not required for this study in accordance with the national legislation and the institutional requirements.

## Author's Note

This study was presented at the 60th Annual meeting of the American Society for Radiation Oncology (ASTRO), October 21–24, 2018, San Antonio, Texas.

## Author Contributions

JC and YK: study concept and design. SK, KK, C-OS, and YK: data acquisition and quality control of data. BL, JC, and YK: data analysis and interpretation. BL and JC: manuscript preparation. JC and YK: manuscript review.

### Conflict of Interest

The authors declare that the research was conducted in the absence of any commercial or financial relationships that could be construed as a potential conflict of interest.
